# Supplementary figures and images for: Shiga Toxin 2 Triggers C3a-Dependent Glomerular and Tubular Injury through Mitochondrial Dysfunction in Hemolytic Uremic Syndrome
Source: Cells. 2022 May 26;11(11):1755. doi: 10.3390/cells11111755 (PMC9179250; doi:10.3390/cells11111755)

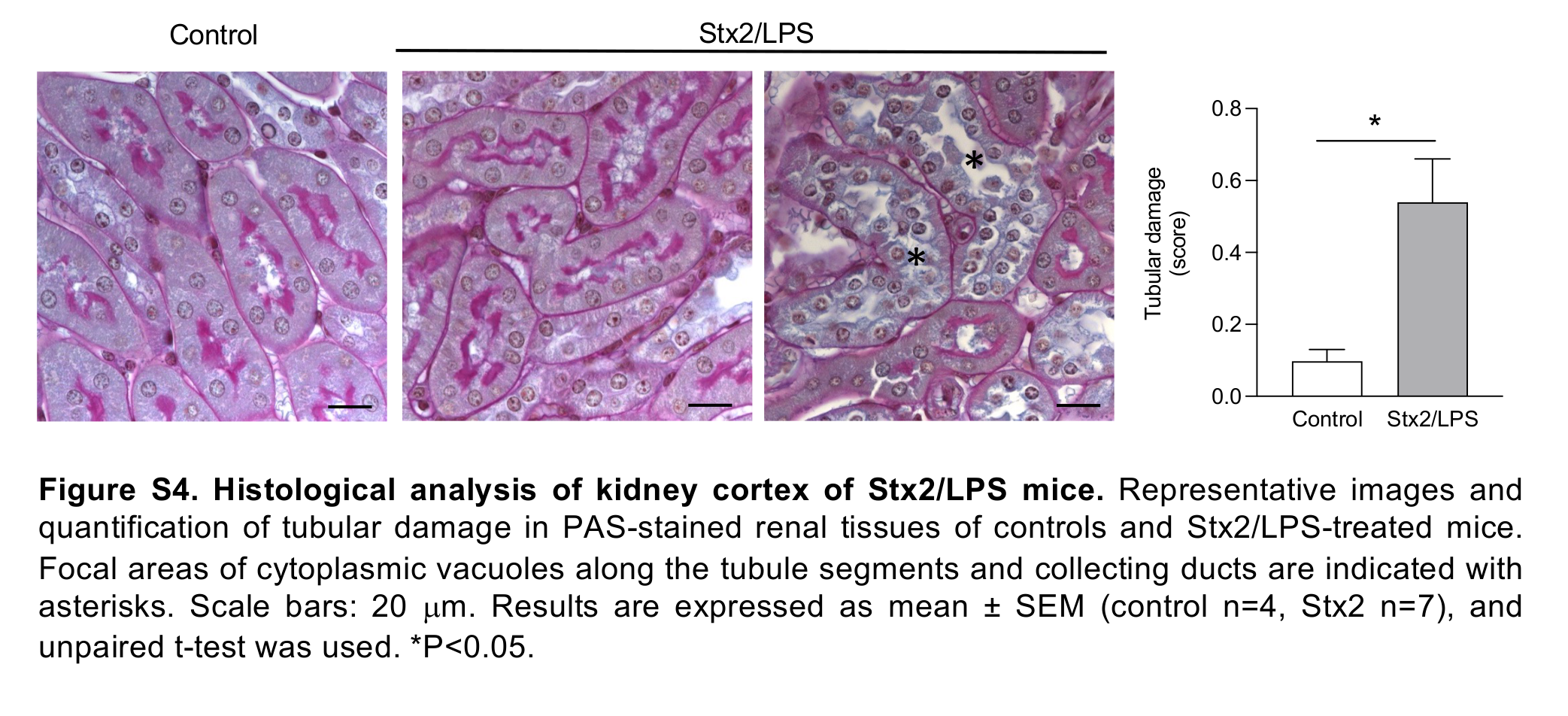

Supplement: Supplementary file 1 [file cells-11-01755-s001.zip › Nuova cartella con elementi/figure S4.tif]

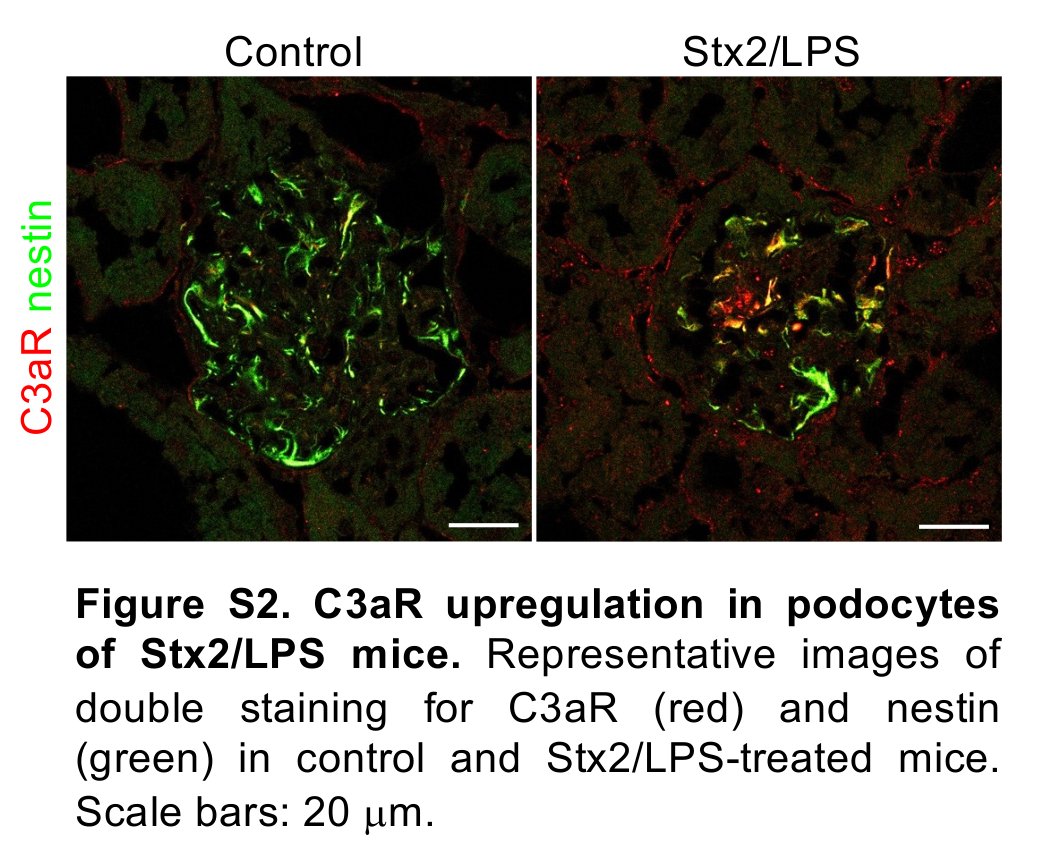

Supplement: Supplementary file 1 [file cells-11-01755-s001.zip › Nuova cartella con elementi/figure S2.tif]

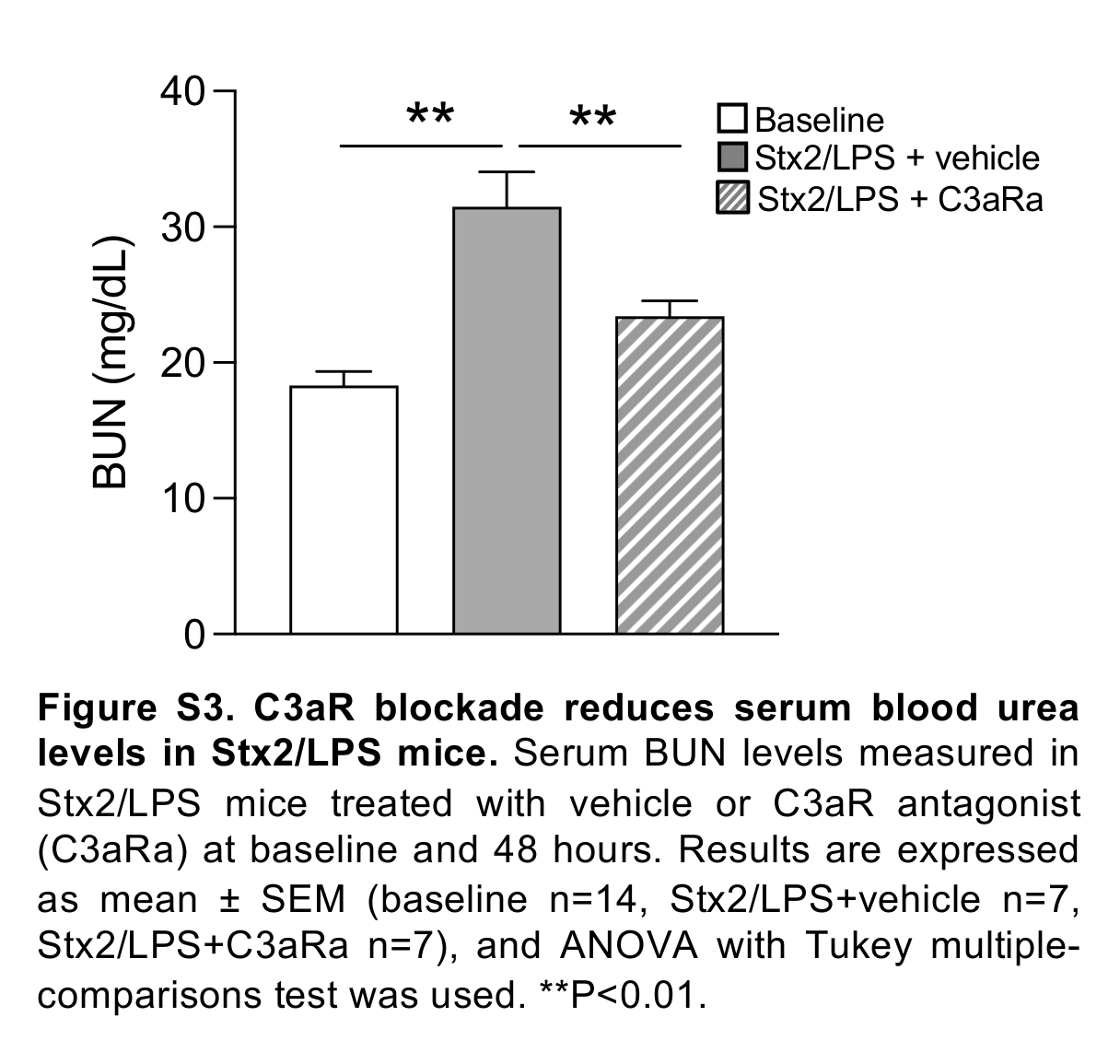

Supplement: Supplementary file 1 [file cells-11-01755-s001.zip › Nuova cartella con elementi/figure S3.tif]

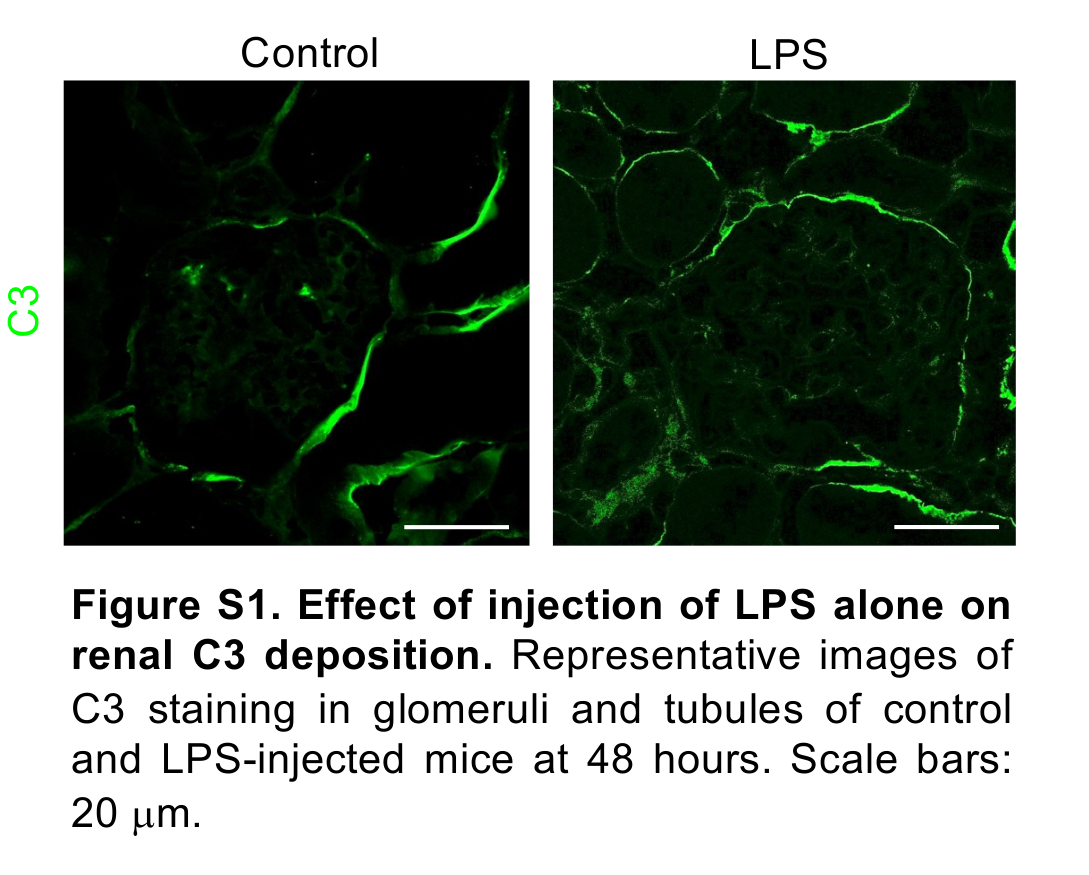

Supplement: Supplementary file 1 [file cells-11-01755-s001.zip › Nuova cartella con elementi/figure S1.tif]
